# Programmatic implementation of depression screening and remote mental health support sessions for persons recently diagnosed with TB in Lima, Peru during the COVID-19 pandemic

**DOI:** 10.1017/gmh.2024.21

**Published:** 2024-04-04

**Authors:** Carmen Contreras, Janeth Santa Cruz, Jerome T. Galea, Alexander L. Chu, Daniela Puma, Lourdes Ramos, Marco Tovar, Jesús Peinado, Leonid Lecca, Salmaan Keshavjee, Courtney M. Yuen, Giuseppe Raviola

**Affiliations:** 1 Socios En Salud Sucursal Peru, Lima, Peru; 2 Harvard Global Health Institute, Harvard University, Cambridge, MA, USA; 3 Department of Global Health and Social Medicine, Harvard Medical School, Boston, MA, USA; 4 School of Social Work, University of South Florida, Tampa, FL, USA; 5 Department of Medical Education, Dell Medical School, The University of Texas at Austin, Austin, TX, USA; 6 Escuela Profesional de Tecnología Médica, Universidad Privada San Juan Bautista, Lima, Peru; 7 Escuela de Medicina, Facultad de Ciencias de la Salud, Universidad Peruana de Ciencias Aplicadas, Lima, Peru; 8 Partners In Health, Boston, MA, USA; 9 Division of Global Health Equity, Brigham and Women’s Hospital, Boston, MA, USA; 10 Department of Psychiatry, Massachusetts General Hospital, Boston, MA, USA

**Keywords:** depression, mental health, tuberculosis, psychological first aid, Peru

## Abstract

**Background:**

Few studies have explored a stepped care model for delivering mental health care to persons with tuberculosis (TB). Here, we evaluated depression screening and remote low-intensity mental health interventions for persons initiating TB treatment in Lima, Peru during the COVID-19 pandemic.

**Methods:**

We used the Patient Health Questionnaire 9 (PHQ-9) to screen participants for depressive symptoms (PHQ-9 ≥ 5). Participants with PHQ-9, 5–14 received remote Psychological First Aid (PFA) or Problem Management Plus (PM+). Participants were reevaluated 6 months after intervention completion. We then compared the change in median PHQ-9 scores before and after intervention completion. Those with PHQ-9 ≥ 15 were referred to higher-level care.

**Findings:**

We found that 62 (45.9%) of the 135 participants had PHQ-9 ≥ 5 at baseline. Then, 54 individuals with PHQ-9, 5–9 received PFA, of which 44 (81.5%) were reevaluated. We observed significant reductions in median PHQ-9 scores from 6 to 2 (*r* = 0.98; *p* < 0.001). Four participants with PHQ-9, 10–14 received PM+ but were unable to be reevaluated. Four participants with PHQ-9 ≥ 15 were referred to higher-level care.

**Conclusions:**

Depressive symptoms were common among persons recently diagnosed with TB. We observed improvements in depressive symptoms 6 months later for most participants who received remote sessions of PFA.

## Graphical abstract

**Figure:**
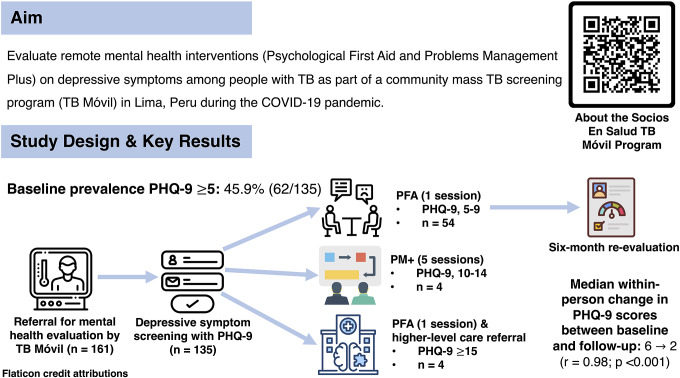

## Impact statement

This report describes one of the first experiences incorporating depression screening and remote mental health support interventions as part of a wider community-based active tuberculosis (TB) screening program. Our findings reaffirm the high prevalence of depressive symptoms among persons recently diagnosed with TB in northern Lima as well as the urgent need to meet and address the psychosocial needs of members of this vulnerable patient population. Importantly, our observations also provide further practical insight into how depression screening and remote mental health interventions may be integrated into existing TB programs, including community-based active TB screening programs.

## Introduction

Tuberculosis (TB) is a debilitating infectious disease caused by *Mycobacterium tuberculosis*, a human pathogen that affects the lungs and other organs, causing significant morbidity and mortality (World Health Organization, 2022b). TB remains a leading cause of mortality due to a single infectious disease after the coronavirus disease (COVID-19) (World Health Organization, 2022a). The World Health Organization (WHO) estimated that in 2022, roughly 10.6 million people acquired TB and 1.3 million – 167,000 of which were HIV positive – died from TB (World Health Organization, 2023). In the Americas region, Peru has one of the highest TB burdens with an estimated annual TB incidence rate of 130 per 100,000 persons per year and is a hotspot for drug-resistant TB (World Health Organization, 2022a).

Mental disorders such as depression are common among persons with TB. It is estimated that about 45% of persons with TB have depression, with prevalence estimates exceeding 50% in persons with multi-drug resistant TB (MDR-TB) (Duko et al., 2020). Similar depression prevalence estimates have been previously reported among persons with TB and MDR-TB in Peru (Vega et al., 2004; Ugarte-Gil et al., 2013). Furthermore, comorbid mental disorders have adverse impacts on TB treatment outcomes. Recent systematic reviews and meta-analyses have reported that persons with TB and depressive symptoms have more than four times the odds of poor TB treatment outcomes compared with those without depressive symptoms (Ruiz-Grosso et al., 2020). Taken together, the current evidence base suggests that addressing comorbid mental disorders such as depression is integral to improving both mental well-being and treatment success rates among those with TB (Sweetland et al., 2018).

Various mental health interventions have demonstrated promise in improving treatment outcomes in persons sick with TB. For example, across three randomized controlled trials, psycho-emotional interventions (including counseling, self-help groups and psychotherapy) were associated with an increased likelihood of achieving successful TB treatment outcomes (pooled RR, 95% CI, 1.37, 1.08–1.73); however, these studies considered all persons with TB and included those without comorbid mental disorders (van Hoorn et al., 2016). A more recently published systematic review by Farooq et al. considered 2 pharmacological and 11 psychosocial interventions for addressing common mental disorders such as depression among persons with TB (*n* = 4,326) in various low- and middle-income countries (LMICs; Farooq et al., 2021). They reported that persons with TB who receive some kind of psychosocial intervention generally have higher TB treatment adherence and cure rates compared with those who do not receive the intervention or when compared with the pre-intervention period. More recently, a large interventional study conducted by Pasha et al. in Pakistan evaluated the implementation of screening for anxiety/depression and subsequent delivery of a series of counseling sessions throughout the TB treatment period among 3,500 persons with TB disease. They found that those who completed at least four sessions had significantly higher rates of completing TB treatment compared with those who completed less than four sessions (Pasha et al., 2021).

Even before the COVID-19 pandemic, there had been a significant interest in implementing remote mental health interventions for various common mental health issues in LMICs (Fu et al., 2020). A recent systematic review and meta-analysis of randomized controlled trials (*n* = 4,104 across 22 trials) found that psychological interventions delivered across various digital modalities (e.g., websites, smartphone apps, computers, audio-devices and text messages) were moderately effective in addressing common mental disorders (pooled Hedges’ *g* = 0.60, 95% CI 0.45–0.75) when compared with control interventions or usual care, and the vast majority of these interventions were used to address depression and substance use disorders (Fu et al., 2020). Since the start of the pandemic, systematic reviews of remote mental health interventions have reported adaptive transitions to implementing synchronous telemental health tools. However, the vast majority of studies were conducted in high-income countries, and commonly cited barriers to scaling up remote mental health interventions in LMICs include a lack of information technology resources and infrastructure in mental health services, socioeconomic inequalities affecting access to remote mental health services and reduced technological literacy (Witteveen et al., 2022).

Despite these challenges, research suggests that designing and delivering remote mental health interventions may be effective in addressing comorbid mental disorders among persons with TB. However, to our knowledge, none has evaluated the implementation of depression screening and delivery of remote mental health interventions embedded within the context of a wider community-based active TB screening program. Furthermore, few studies have reported the use of a stepped care model for allocating different low-intensity mental health support interventions based on different depressive symptom severities at the time of initial screening (Bower and Gilbody, 2005; Walker et al., 2018). Thus, the aim of this study was to evaluate remote low-intensity mental health interventions, specifically Psychological First Aid and Problems Management+, on depressive symptoms among people initiating treatment for pulmonary TB as part of a community-based active TB screening program in Lima, Peru during the COVID-19 pandemic.

## Methods

### Study design and context

We conducted a secondary analysis of data collected as part of a mental health program run by the nonprofit organization Socios En Salud based in Lima, Peru. This program screened persons with active TB for depressive symptoms and subsequently provided low-intensity mental health interventions during their TB treatment period between 2019 and 2021. This mental health program was incorporated as part of a wider community-based active TB screening program called “TB Móvil” (TB-M), which SES first implemented in collaboration with the Ministry of Health (MINSA) of Peru in 2019 to identify persons with active TB across various districts of Metropolitan Lima (Yuen et al., 2021; Galea et al., 2022). Persons diagnosed with TB through the TB-M program were referred to participate in the SES mental health program. The mental health program was implemented remotely in parallel to the TB-M program between September 2020 and June 2021 during the early phases of the COVID-19 pandemic.

### Intervention procedures

#### Participant enrollment and data collection

Participants were eligible for depressive symptom screening if they satisfied the following inclusion criteria: individuals were recently diagnosed with TB through the TB-M program and had subsequently initiated treatment; were referred by TB-M program staff members for further mental health evaluation; were 18 years or older; and were residents of communities and districts of northern Lima that were a part of the TB-M program catchment area. SES psychologists contacted individuals who were referred by the TB-M program staff by telephone and then invited them to be screened for depressive symptoms within 30 days of initiating TB treatment. Those who did not start TB treatment during this period were excluded. Participant sociodemographic (e.g., age, sex, highest level of educational attainment and region of birth), clinical and microbiological data (e.g., TB disease diagnosis, sputum smear microscopy status, GeneXpert results and rifampin resistance status) were obtained from the TB-M program’s database.

#### Data preparation and handling

For this secondary data analysis, we accessed non-identifiable information on TB-M program participants from the SES informatics system. To describe the sociodemographic and clinical characteristics, we considered the following variables among all participants evaluated for depressive symptoms: age, sex, highest educational level attained, region of birth, BK results, GeneXpert results, chest radiography status and rifampin resistance status. We considered the age of the participants as a continuous variable. The highest level of education attained was categorized into three groups: primary, secondary and postsecondary. We defined region of birth as being born inside or outside the Lima region. Clinical variables like BK and GeneXpert results were considered as binary variables – negative or positive.

The main variables analyzed in this study were depressive symptoms at baseline and follow-up as well as the type of remote mental health intervention provided by mental health professionals.

#### Depressive symptom screening and definitions

SES psychologists interviewed participants using the validated Spanish version of the Patient Health Questionnaire 9 (PHQ-9), a depression screening instrument widely used in clinical practice and research (Calderón et al., 2012). The score evaluates the number and frequency of nine depressive symptoms and ranges from 0 (i.e., experiencing no depressive symptoms none of the time) to 27 (i.e., experiencing all depressive symptoms nearly every day). The PHQ-9 uses different score ranges to classify different depressive symptom severities. They include minimal (PHQ-9 ≤ 4), mild (PHQ-9, 5–9), moderate (PHQ-9, 10–14), moderately severe (PHQ-9, 15–19) and severe (PHQ-9 score ≥20) (Kroenke et al., 2001).

#### Mental health interventions

The mental health interventions offered as part of the SES mental health program were originally developed before the pandemic and subsequently underwent revisions at the onset of the pandemic. Here, we describe the mental health interventions that were delivered during the early phases of the pandemic period specifically. Between September 2020 and June 2021, remote mental health support sessions were offered to participants identified with signs and symptoms of depression. Those with PHQ-9 scores of 5–9 received one session of Psychological First Aid (PFA), and those with PHQ-9 scores from 10 to 14 received five support sessions of Problem Management Plus (PM+) (Dawson et al., 2015). People with PHQ-9 scores ≥15 received one session of PFA before being promptly referred for higher-level mental health care at public health care institutions. The remote PFA and PM+ support sessions and referral process for patients with severe depressive symptoms are further described in detail below.

##### Psychological first aid

The remote PFA sessions provided basic psychological support in emergent and stressful situations. The primary aim of the remote PFA support sessions was to help participants restore their emotional balance according to three principles: (1) observe the person’s problem, needs, and possible solutions; (2) listen carefully to the person and help him/her feel supported and address his/her basic needs and (3) connect the individual to public or private mental health institutions if further specialized care was needed (International Federation of Red Cross and Red Crescent Societies, 2020). This intervention was administered on an individual basis by SES psychologists *via* a telephone call, consisting of a single session lasting approximately 45 min.

##### Problem management plus

PM+ is a low-intensity, transdiagnostic psychological intervention recommended by the WHO in treating common mental disorders in many resource-limited settings (World Health Organization, 2016; Hamdani et al., 2018). Previous studies have shown that it is effective in reducing symptoms of anxiety and depression (Rahman et al., 2016; Hamdani et al., 2018). Its primary advantage is that it can be delivered widely by trained nonspecialists such as community health workers, volunteers, and psychology students. The PM+ intervention has previously been adapted for use in the general Peruvian population (Coleman et al., 2021). The intervention consisted of five remote 90-min sessions that were delivered on an individual basis every week. In the first session, participants were oriented and motivated to participate and receive psychoeducation and learn basic stress management and control strategies. In the second session, participants learned problem-solving techniques for life problems and were introduced to behavioral activation techniques. In the third and fourth sessions, they were introduced to techniques for strengthening social support and continued to practice problem-solving techniques, behavioral activation procedures and relaxation exercises. In the last session, all learned strategies were reviewed and demonstrated by participants as a means of assessing understanding for future use and application.

### Referral process and criteria for further specialized mental health care

SES psychologists referred participants with PHQ-9 ≥ 15 for higher-level mental health care at public health care institutions. The referral process included identifying public health care facilities closest to the participant’s home, contacting the local facility and arranging appropriate follow-up to ensure that the referral process was successful. In areas without access to mental health facilities, participants were referred to a nearby health care center instead. Those experiencing mental health problems other than depression were referred to specialized public mental health services operated by the MINSA of Peru.

#### Depression reevaluation

Among those who had received and completed remotely administered sessions of PFA or PM+, SES psychologists contacted those same participants 6 months later and invited them to be reevaluated for depressive symptoms using the PHQ-9 questionnaire.

#### Data collection and statistical analysis

SES psychologists uploaded data collected from participants to the SES electronic data system. These included PHQ-9 scores at the time of enrollment/baseline and at the time of follow-up, type of remote mental health support sessions received (PFA or PM+), and whether participants were referred to primary care facilities or public health centers for higher-level mental health care. Continuous variables of characteristics of people with TB were reported as medians with interquartile ranges (IQR), and categorical variables were reported as frequencies with percentages. We reported the overall proportion of people initiating treatment for TB with PHQ-9 ≥ 5 at the time of study enrollment. For participants who were reevaluated, we used the Wilcoxon’s paired signed-rank test to compare the within-person change in median PHQ-9 scores between baseline and the 6-month follow-up. We reported the relevant effect size (“*r*”) estimate and accompanying p-value (Rosenthal, 1994; Kerby, 2014). All statistical analyses were conducted using Stata/SE 17.0 (Stata Corp., College Station, TX) with a significance level of 0.05.

## Results

**Figure 1.**
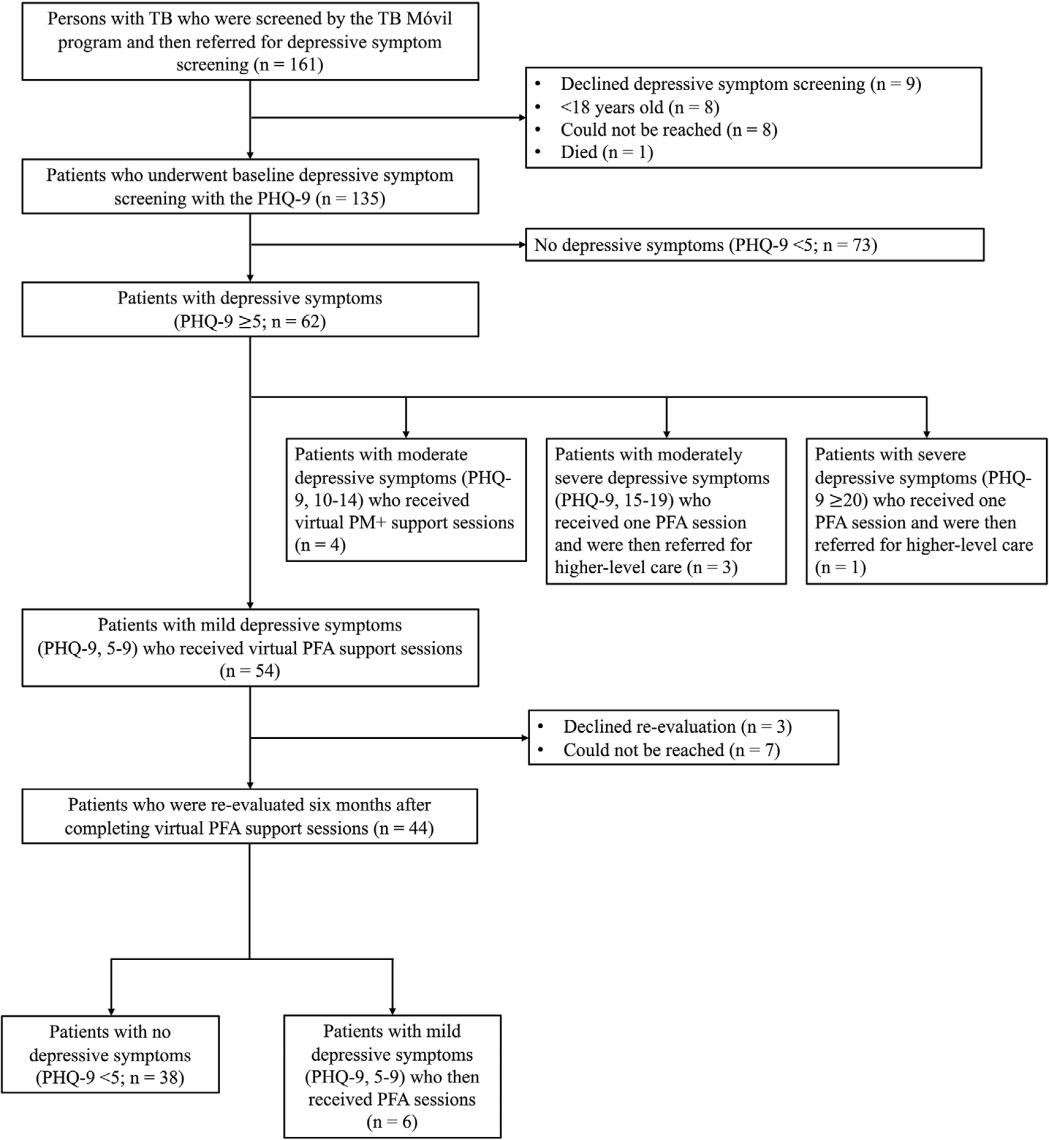
Study flowchart.

During the pandemic, we identified a total of 161 individuals who were referred for mental health evaluation after being assessed by the TB-M program (Figure 1). After excluding 26 participants, a total of 135 (83.9%) eligible participants underwent depressive symptom screening at baseline. Among all persons with TB who had undergone depressive symptom screening at baseline, the median age was 38.9 years (IQR: 28.4 years) and the majority were male (76 of 135 participants, 56.3%) (Table 1). Most participants were born in the Lima region (101 of 130 participants, 77.7%) and completed secondary education (91 of 129 participants, 70.5%). Microbiologically, 106 (81.5%) of 130 participants were tested for TB using sputum samples. Of those who had available GeneXpert MTB complex results (*n* = 100), 90 (90.0%) were positive; 10 (11.1%) of 90 participants with information on rifampicin resistance were resistant to rifampin.

**Table 1.** Baseline participant characteristics, by baseline depressive symptom status (*N* = 135)

Characteristics		Baseline depressive symptom status, no. % (column)		
	Total no. (%), *N* = 135^a^	PHQ-9 scores < 5 (*n* = 73)	PHQ-9 scores ≥ 5 (*n* = 62)	*p*-Value
Age, years, median (IQR) (*n* = 135)	135 (100.0)	41.6 (30.5)	37.2 (22.0)	0.581
Sex (*n* = 135)				0.055
Male	76 (56.3)	47 (64.4)	29 (46.8)	
Female	59 (43.7)	26 (35.6)	33 (53.2)	
Highest educational level achieved (*n* = 129)				
Primary or less	24 (17.8)	14 (20.6)	10 (16.4)	0.816
Secondary	91 (70.5)	46 (67.7)	45 (73.8)	
Post-secondary	14 (10.9)	8 (11.8)	6 (9.8)	
Region of birth (*n* = 130)				0.035
From Lima	101 (77.7)	50 (70.4)	51 (86.4)	
Outside of Lima	29 (22.3)	21 (29.6)	8 (13.6)	
TB diagnosis methodology (*n* = 130)				0.652
Microbiological confirmation	106 (81.5)	54 (79.4)	52 (83.9)	
Clinical/radiological criteria	24 (18.5)	14 (20.6)	10 (16.1)	
BK results (*n* = 100)				0.159
Negative	53 (53.0)	33 (60.0)	20 (44.4)	
Positive	47 (47.0)	22 (40.0)	25 (55.6)	
GeneXpert MTB complex detection status (*n* = 100)				0.021
Negative/not detected	10 (10.0)	9 (16.4)	1 (2.2)	
Positive/detected	90 (90.0)	46 (83.6)	44 (97.8)	
Rifampin resistance status^b^ (*n* = 90)				0.076
Sensitive	49 (54.4)	25 (54.3)	24 (54.5)	
Resistant	31 (34.4)	19 (41.3)	12 (27.3)	
Indeterminant	10 (11.1)	2 (4.3)	8 (18.2)	

a Total number may be less than 135 due to missing data.

b Numbers and percentages reported only among participants with a positive GeneXpert test result.

Among 135 participants who underwent screening for depression at baseline, 62 (45.9%) had PHQ-9 ≥ 5 (Table 1). Persons with TB and PHQ-9 ≥ 5 at baseline tended to be younger compared with those with PHQ-9 < 5; albeit the comparison was not statistically significant (median age and IQR for participants with depressive symptoms vs. without at baseline: 37.2 years [22.0 years] vs. 41.6 years [30.5 years], *p* = 0.581) (Table 1). Furthermore, we did not find any statistically significant difference in the proportion of participants with PHQ-9 ≥ 5 at baseline neither by sex (*p* = 0.055) nor by education level (*p* = 0.816; Table 2). However, those with PHQ-9 ≥ 5 at baseline were more likely to be born within Lima compared with those with PHQ-9 scores <5 (PHQ-9 ≥ 5 vs. <5 for the region of birth within Lima: 50 [70.4%] vs. 51 [86.4%], *p* = 0.035). We did not find a statistically significant difference in baseline depression status for those who had microbiological confirmation of their TB compared with those diagnosed based on clinical/radiological findings (*p* = 0.652).

**Table 2.** Breakdown of depressive symptom severity at baseline among persons with TB

Baseline depressive symptom severity	PHQ-9 score range	No. (%)
None/minimal	0–4	73 (54.1)
Mild	5–9	54 (40.0)
Moderate	10–14	4 (3.0)
Moderately severe	15–19	3 (2.2)
Severe	≥20	1 (0.7)
**Total**		**135 (100.0)**

Of the 62 participants who were found to have PHQ-9 ≥ 5 at baseline, almost all had PHQ-9, 5–9 (54 [87.1%]); 4 (6.5%) participants had PHQ-9, 10–14; 3 (4.8%) had PHQ-9, 15–19 and 1 (1.6%) had PHQ-9 ≥ 20 (Table 2 and Figure 1). Among the 54 participants who were found to have PHQ-9, 5–9, 44 (81.5%) were reevaluated 6 months after completing remote PFA support sessions. The majority of participants reevaluated 6 months later no longer had clinically significant depressive symptoms, as evidenced by PHQ-9 scores <5 (*n* = 38 (86.4%)); only 6 (13.6%) had PHQ-9, 5–9. We observed a statistically significant reduction in the within-person change in the median PHQ-9 scores at baseline and during the 6-month follow-up after completing the remote PFA support sessions (median PHQ-9 score and IQR at baseline and at follow-up: 6 [3] and 2 [3], respectively, (*r* = 0.98; *p* < 0.001; Table 3). Remote PM+ support sessions were delivered to four participants who were initially found to have PHQ-9, 10–14; however, they all refused reevaluation 6 months later, and, therefore, a comparison could not be made. All participants who were found to have PHQ-9 ≥ 15 successfully received a single remote session of PFA and were immediately referred to higher-level mental health care.

**Table 3.** Comparisons of median PHQ-9 scores at baseline and 6 months following completion of remote PFA support sessions in persons with TB (*n* = 44)

Baseline PHQ-9				PHQ-9 at reevaluation (6-month follow-up)				Change in median PHQ-9	
Median	Min	Max	IQR	Median	Min	Max	IQR	Effect size (*r*)	*p*-Value
6	5	9	3	2	0	5	3	0.98	<0.001

## Discussion

During the COVID-19 pandemic, we found that almost half of all persons recently diagnosed with TB as part of a community-based active TB screening program exhibited depressive symptoms (PHQ-9 ≥ 5). Furthermore, our findings also indicate that implementing a stepped care model for mental health screening and care delivery was associated with overall improvements in median PHQ-9 scores 6 months later.

Overall, we found that 45.9% of all persons with TB in our sample had depressive symptoms at the time of TB treatment initiation. We recognize that this prevalence estimate was determined using a liberal PHQ-9 cutoff score of 5. Nonetheless, our prevalence estimate is much higher than that recently reported in the general Peruvian population during the pandemic period (~20%) (Zegarra-López et al., 2022). Our estimate is consistent with the pooled depressive symptom prevalence estimate among persons with TB (Duko et al., 2020). In our program, most participants with depressive symptoms exhibited mild depression, defined by PHQ-9, 5–9 (54/62 [87.1%]). Most were contacted by SES psychologists within the first 30 days following TB diagnosis, a period that also coincides with the initiation of TB treatment. The high prevalence at baseline may be related to an increased incidence of new depressive symptoms or an acute worsening of pre-existing depression or some other unassessed mental disorders within our study population. While our data limits our ability to differentiate between these two possibilities, the high prevalence of depressive symptoms may be due to a variety of acute psychosocial stressors related to receiving a TB diagnosis and/or transitioning to receiving TB treatment (Sweetland et al., 2017). In particular, the perceived stigma associated with a TB diagnosis is notably high among persons with TB, and its sequelae (e.g., discrimination) are common means by which persons with TB may develop and experience depressive symptoms (Lee et al., 2017; Sweetland et al., 2017; Mohammedhussein et al., 2020; Chen et al., 2023). Thus, our findings suggest that the integration of early depression screening within TB screening programs could be useful in identifying a large sample of patients who may benefit from concurrent mental health care during their TB treatment, especially around the time of TB diagnosis and initiation of TB treatment.

Previous studies addressing comorbid mental and health concerns among persons with TB disease have primarily focused on delivering mental health interventions to those who screen positive for depressive symptoms, regardless of their symptom severity at the time of initial screening (Farooq et al., 2021). However, few studies have implemented a stepped care model of first screening for mental disorders and subsequently delivering severity-appropriate mental health interventions. In Nepal, Walker et al. conducted a feasibility and acceptability pilot study for a psychosocial support package among patients with MDR-TB (Walker et al., 2018). Their package involved providing all patients with information and educational materials and initially screening them for symptoms of anxiety and depression using the Johns Hopkins Symptom Checklist. For those who screened positive for either anxiety or depression, they were subsequently referred for depressive symptom screening using the PHQ-9. Those who had PHQ-9 scores less than 10 were rescreened on a monthly frequency. Those who had PHQ-9, 10–19 received a series of counseling sessions based on behavioral activation originally evaluated in India for treating depression. Those who had PHQ-9 > 19 or who expressed suicidal intent were referred for higher-level psychiatric and medical care. Although this study concluded that, overall, it was feasible to design and implement a stepped care model for mental health care within a National Tuberculosis Program, it suffered a couple of key limitations, including (a) utilization of a complex two-step screening system that likely resulted in fewer number of patients receiving the counseling intervention and (b) inability to evaluate the potential impact of the mental health intervention on changes in depression and anxiety severity in relation to the time of initial screening. The present study utilized the PHQ-9 as the only standardized screening tool and implemented a stepped care model with different severity-appropriate virtual mental health support sessions. Although we were unable to reevaluate enough participants who received remote PM+ sessions, we were able to reevaluate a high proportion (~80%) of those who completed remote PFA sessions and gain a sense of the possible mental health impact associated with providing severity-specific mental health interventions.

More broadly speaking, our findings and programmatic experience are consistent with overall global patterns of shifting toward remote means of delivering mental health care and support at the onset of the COVID-19 pandemic. This shift has been described by a recent umbrella review of 38 systematic reviews of studies on remote mental health interventions and care delivery during the pandemic (Witteveen et al., 2022). In particular, there was a greater shift toward synchronous modalities of remote mental health care delivery (e.g., use of videoconferencing platforms and telephone calls) than asynchronous means (e.g., self-help apps, websites or digital tools) (Witteveen et al., 2022). This shift is also consistent with our programmatic approach with the use of telephone calls and videoconferencing means to deliver remote PFA sessions and PM+ sessions. However, despite these adaptive changes, these reviews also highlighted limited access to remote mental health care and services among members of vulnerable populations and communities as a chief barrier and disparity; this included a paucity of studies reporting on remote mental health interventions from LMICs, particularly during the early phase of the pandemic (Witteveen et al., 2022). Our study adds to a growing need for research that evaluates innovative and integrated ways of meeting the psychosocial needs of vulnerable patient populations like those with TB in resource-limited settings.

Our programmatic experience and findings have implications for integrating mental health care and TB care. We show that low-intensity mental health interventions, such as PFA and PM+ support sessions, can be administered virtually to persons recently diagnosed with TB during the pandemic era. This type of care delivery modality is in line with the wider and accelerated shifts toward adapting and utilizing telehealth-based technologies during the pandemic (Moreno et al., 2020; Witteveen et al., 2022). Low-intensity psychosocial interventions such as PFA are first-line psychosocial interventions that can be administered in high-stress mental health crises and delivered by trained nonspecialist personnel. This may greatly expand service coverage in settings with limited resources and fewer formally trained mental health professionals (Pollock et al., 2020); these lessons may also be applicable in high-income settings. Furthermore, integrated TB and mental health screening in high-risk populations, settings or communities offers an opportunity to detect a higher number of individuals with both TB and mental health issues. This may be particularly important during times when acute psychosocial stressors and TB-associated stigma are likely to be most severe.

Our study has several notable limitations. First, although we observed statistically significant reductions in the median PHQ-9 scores from baseline to 6 months following completion of PFA sessions, we are limited in our ability to infer to what extent the mental health interventions may have contributed to these reductions. On the one hand, remote PFA sessions could have improved depressive symptoms by providing immediate psychosocial support to persons with newly diagnosed TB during what is often a significant and traumatic life event and transition (Hermosilla et al., 2023). On the other hand, we also considered other plausible explanations, which include, but are not limited to, natural improvements in depressive symptoms with time, improvements in TB disease because of ongoing treatment, the beneficial impacts of other unmeasured psychosocial and/or clinical factors (i.e., residual confounding) or a combination of these factors. In a similar vein, we were unable to compare changes in median PHQ-9 scores over time with a control group, as every participant who had PHQ-9 ≥ 5 at baseline was offered mental health support. Second, we were unable to reevaluate slightly less than 20% of those who had PHQ-9 scores of 5–9 at baseline 6 months after completing PFA support sessions. Assuming those with higher PHQ-9 scores are less likely to follow-up, this could have led to an overestimation of the change in median PHQ-9 scores between baseline and follow-up. Finally, we were unable to assess the association between completing the mental health interventions and TB treatment outcomes, including known mediators such as treatment adherence. Previous studies have demonstrated a positive correlation between emotional support during TB treatment and improved treatment adherence and success rates (Ruiz-Grosso et al., 2020). Therefore, future mental health interventions could include regular, monthly follow-up depression assessments throughout the TB treatment period, particularly at the start of TB treatment when depressive symptoms are likely to be most severe.

In conclusion, we found that depressive symptoms were common among people with TB who were identified by a community-based active TB screening program. We also observed significant improvements in depressive symptoms 6 months later among most participants who received remote sessions of PFA. Future studies are needed to rigorously evaluate the feasibility and utility of frequent depression assessments during the TB treatment period as well as the impact of severity-appropriate, low-intensity psychosocial interventions on TB treatment outcomes regarding cost, programmatic scalability, and acceptability among persons with TB.

## Data Availability

The anonymized version of the data analyzed in this study is available upon request.

## References

[r1] Bower P and Gilbody S (2005) Stepped care in psychological therapies: Access, effectiveness and efficiency. Narrative literature review. British Journal of Psychiatry 186(1), 11–17. 10.1192/bjp.186.1.11 15630118

[r2] Calderón M, Gálvez-Buccollini JA, Cueva G, Ordoñez C, Bromley C and Fiestas F (2012) Validación de la versión peruana del PHQ-9 Para el diagnóstico de depresión. Revista Peruana de Medicina Experimental y Salud Pública 29(4), 578–579. 10.1590/S1726-46342012000400027 23338650

[r3] Chen X, Chen Y, Zhou L and Tong J (2023) The role of self-esteem as moderator of the relationship between experienced stigma and anxiety and depression among tuberculosis patients. Scientific Reports 13(1), 6889. 10.1038/s41598-023-34129-4 37105982 PMC10134698

[r4] Coleman SF, Mukasakindi H, Rose AL, Galea JT, Nyirandagijimana B, Hakizimana J, Bienvenue R, Kundu P, Uwimana E, Uwamwezi A, Contreras C, Rodriguez-Cuevas FG, Maza J, Ruderman T, Connolly E, Chalamanda M, Kayira W, Kazoole K, Kelly KK, Wilson JH, Houde AA, Magill EB, Raviola GJ and Smith SL (2021) Adapting problem management plus for implementation: Lessons learned from public sector settings across Rwanda, Peru, Mexico and Malawi. Intervention (Amstelveen, Netherlands) 19(1), 58–66. 34642580 PMC8503941

[r5] Dawson KS, Bryant RA, Harper M, Tay AK, Rahman A, Schafe A and van Ommeren M (2015) Problem management plus (PM+): A WHO transdiagnostic psychological intervention for common mental health problems. World Psychiatry 14(3), 354–357. 10.1002/wps.20255 26407793 PMC4592660

[r6] Duko B, Bedaso A and Ayano G (2020) The prevalence of depression among patients with tuberculosis: A systematic review and meta-analysis. Annals of General Psychiatry 19, 30. 10.1186/s12991-020-00281-8 32419837 PMC7206806

[r7] Farooq S, Tunmore J and Comber R (2021) Pharmacological or non-pharmacological interventions for treatment of common mental disorders associated with tuberculosis: A systematic review. Chronic Respiratory Disease 18, 147997312110039. 10.1177/14799731211003937 PMC8082988 33896235

[r8] Fu Z, Burger H, Arjadi R and Bockting CLH (2020) Effectiveness of digital psychological interventions for mental health problems in low-income and middle-income countries: A systematic review and meta-analysis. Lancet. Psychiatry 7(10), 851–864. 10.1016/S2215-0366(20)30256-X 32866459 PMC7455253

[r9] Galea JT, Puma D, Tzelios C, Valdivia H, Millones AK, Jiménez J, Brooks MB, Yuen CM, Lecca L, Becerra MC and Keshavjee S (2022) A structured community engagement strategy to support uptake of TB active case-finding. Public Health Action 12(1), 18–23. 10.5588/pha.21.0059 35317536 PMC8908875

[r10] Hamdani SU, Ahmed Z, Sijbrandij M, Nazir H, Masood A, Akhtar P, Amin H, Bryant RA, Dawson K, van Ommeren M, Rahman A and Minhas FA (2018) Correction to: Problem management plus (PM+) in the management of common mental disorders in a specialized mental healthcare facility in Pakistan; study protocol for a randomized controlled trial. International Journal of Mental Health Systems 12(1), 53. 10.1186/s13033-018-0231-1 30275875 PMC6158843

[r11] Hermosilla S, Forthal S, Sadowska K, Magill EB, Watson P and Pike KM (2023) We need to build the evidence: A systematic review of psychological first aid on mental health and well-being. Journal of Traumatic Stress 36(1), 5–16. 10.1002/jts.22888 36300605 PMC10624106

[r12] International Federation of Red Cross and Red Crescent Societies (2020) *Remote Psychological First Aid During the COVID-19 Outbreak Interim Guidance - March 2020* (Technical Guidelines). Available at https://reliefweb.int/report/world/remote-psychological-first-aid-during-covid-19-outbreak-interim-guidance-march-2020 (accessed March 22, 2023).

[r13] Kerby DS (2014) The simple difference formula: An approach to teaching nonparametric correlation. Comprehensive Psychology 3, 11.IT.3.1. 10.2466/11.IT.3.1

[r14] Kroenke K, Spitzer RL and Williams JB (2001) The PHQ-9: Validity of a brief depression severity measure. Journal of General Internal Medicine 16(9), 606–613. https://doi.oeg/10.1046/j.1525-1497.2001.016009606.x 11556941 10.1046/j.1525-1497.2001.016009606.x PMC1495268

[r15] Lee L, Tung H, Chen S and Fu C (2017) Perceived stigma and depression in initially diagnosed pulmonary tuberculosis patients. Journal of Clinical Nursing 26(23–24), 4813–4821. 10.1111/jocn.13837 28370819

[r16] Mohammedhussein M, Hajure M, Shifa JE and Hassen TA (2020) Perceived stigma among patient with pulmonary tuberculosis at public health facilities in Southwest Ethiopia: A cross-sectional study. PLoS One 15(12), e0243433. 10.1371/journal.pone.0243433 33290413 PMC7731994

[r17] Moreno C, Wykes T, Galderisi S, Nordentoft M, Crossley N, Jones N, Cannon M, Correll CU, Byrne L, Carr S, Chen EYH, Gorwood P, Johnson S, Kärkkäinen H, Krystal JH, Lee J, Lieberman J, López-Jaramillo C, Männikkö M, Phillips MR, Uchida H, Vieta E, Vita A and Arango C (2020) How mental health care should change as a consequence of the COVID-19 pandemic. Lancet Psychiatry 7(9), 813–824. 10.1016/S2215-0366(20)30307-2 32682460 PMC7365642

[r18] Pasha A, Siddiqui H, Ali S, Brooks MB, Maqbool NR and Khan AJ (2021) Impact of integrating mental health services within existing tuberculosis treatment facilities. Medicine Access @ Point of Care 5, 23992026211011314. 10.1177/23992026211011314 36204497 PMC9413619

[r19] Pollock A, Campbell P, Cheyne J, Cowie J, Davis B, McCallum J, McGill K, Elders A, Hagen S, McClurg D, Torrens C and Maxwell M (2020) Interventions to support the resilience and mental health of frontline health and social care professionals during and after a disease outbreak, epidemic or pandemic: A mixed methods systematic review. Cochrane Database of Systematic Reviews 11, CD013779. 10.1002/14651858.CD013779 33150970 PMC8226433

[r20] Rahman A, Riaz N, Dawson KS, … Farooq S (2016) Problem management plus (PM+): Pilot trial of a WHO transdiagnostic psychological intervention in conflict-affected Pakistan. World Psychiatry 15(2), 182–183. 10.1002/wps.20312 27265713 PMC4911784

[r21] Rosenthal R (1994) Parametric measures of effect size. In Cooper H and Hedges LV (eds.), The Handbook of Research Synthesis. New York: Russell Sage Foundation, pp. 231–244.

[r22] Ruiz-Grosso P, Cachay R, de la Flor A, Schwalb A and Ugarte-Gil C (2020) Association between tuberculosis and depression on negative outcomes of tuberculosis treatment: A systematic review and meta-analysis. PLoS One 15(1), e0227472. 10.1371/journal.pone.0227472 31923280 PMC6953784

[r23] Sweetland AC, Jaramillo E, Wainberg ML, Chowdhary N, Oquendo MA, Medina-Marino A and Dua T (2018) Tuberculosis: An opportunity to integrate mental health services in primary care in low-resource settings. Lancet. Psychiatry 5(12), 952–954. 10.1016/S2215-0366(18)30347-X 30241700 PMC6489124

[r24] Sweetland AC, Kritski A, Oquendo MA, … Wainberg ML (2017) Addressing the tuberculosis–depression syndemic to end the tuberculosis epidemic. International Journal of Tuberculosis and Lung Disease 21(8), 852–861. 10.5588/ijtld.16.0584 PMC5759333 28786792

[r25] Ugarte-Gil C, Ruiz P, Zamudio C, Canaza L, Otero L, Kruger H and Seas C (2013) Association of major depressive episode with negative outcomes of tuberculosis treatment. PLoS One 8(7), e69514. 10.1371/journal.pone.0069514 23922728 PMC3726639

[r26] van Hoorn R, Jaramillo E, Collins D, Gebhard A and van den Hof S (2016) The effects of psycho-emotional and socio-economic support for tuberculosis patients on treatment adherence and treatment outcomes – a systematic review and meta-analysis. PLoS One 11(4), e0154095. 10.1371/journal.pone.0154095 27123848 PMC4849661

[r27] Vega P, Sweetland A, Acha J, Castillo H, Guerra D, Smith Fawzi MC and Shin S (2004) Psychiatric issues in the management of patients with multidrug-resistant tuberculosis. International Journal of Tuberculosis and Lung Disease 8(6), 749–759. 15182146

[r28] Walker IF, Khanal S, Hicks JP, Lamichhane B, Thapa A, Elsey H, Baral SC and Newell JN (2018) Implementation of a psychosocial support package for people receiving treatment for multidrug-resistant tuberculosis in Nepal: A feasibility and acceptability study. PLoS One 13(7), e0201163. 10.1371/journal.pone.0201163 30048495 PMC6062069

[r29] Witteveen AB, Young S, Cuijpers P, Ayuso-Mateos JL, Barbui C, Bertolini F, Cabello M, Cadorin C, Downes N, Franzoi D, Gasior M, John A, Melchior M, McDaid D, Palantza C, Purgato M, Van der Waerden J, Wang S and Sijbrandij M (2022) Remote mental health care interventions during the COVID-19 pandemic: An umbrella review. Behaviour Research and Therapy 159, 104226. 10.1016/j.brat.2022.104226 36410111 PMC9661449

[r30] World Health Organization (2016) Problem Management Plus (PM+): Individual Psychological Help for Adults Impaired by Distress in Communities Exposed to Adversity. Geneva: World Health Organization. Available at https://www.who.int/publications/i/item/WHO-MSD-MER-16.2 (accessed March 22, 2023).

[r31] World Health Organization (2022a) Global Tuberculosis Report 2022. Geneva: World Health Organization. Available at https://www.who.int/teams/global-tuberculosis-programme/tb-reports/global-tuberculosis-report-2022 (accessed March 22, 2023).

[r32] World Health Organization (2022b) Tuberculosis Fact Sheet. Geneva: World Health Organization. Available at https://www.who.int/news-room/fact-sheets/detail/tuberculosis (accessed March 22, 2023).

[r33] World Health Organization (2023) Global Tuberculosis Report 2023. Geneva: World Health Organization. Available at https://www.who.int/teams/global-tuberculosis-programme/tb-reports/global-tuberculosis-report-2023 (accessed December 3, 2023).

[r34] Yuen CM, Puma D, Millones AK, Galea JT, Tzelios C, Calderon RI, Brooks MB, Jimenez J, Contreras C, Nichols TC, Nicholson T, Lecca L, Becerra MC and Keshavjee S (2021) Identifying barriers and facilitators to implementation of community-based tuberculosis active case finding with mobile X-ray units in Lima, Peru: A RE-AIM evaluation. BMJ Open 11(7), e050314. 10.1136/bmjopen-2021-050314 PMC8264873 34234000

[r35] Zegarra-López AC, Florentino-Santisteban B, Flores-Romero J, Delgado-Tenorio A and Cernades-Ames A (2022) A cross-sectional study on the prevalence of depressive symptoms and its associated sociodemographic factors in Peru during the COVID-19 pandemic. International Journal of Environmental Research and Public Health 19(21), 14240. 10.3390/ijerph192114240 36361118 PMC9654240

