# Supplementary material for: Programmatic implementation of depression screening and remote mental health support sessions for persons recently diagnosed with TB in Lima, Peru during the COVID-19 pandemic
Source: Glob Ment Health (Camb). 2024 Apr 4;11:e59. doi: 10.1017/gmh.2024.21 (PMC11094547; doi:10.1017/gmh.2024.21)
Supplement: Peer review history

# Editor report: Recommendation: Programmatic implementation of depression screening and remote mental health support sessions for persons recently diagnosed with TB in Lima, Peru during the COVID-19 pandemic — R0/PR2

**DOI:** 10.1017/gmh.2024.21.pr2

**Affiliations:** University of KwaZulu-Natal, South Africa

Kindly attend to the reviewers comments and resubmit

# Editor report: Decision: Programmatic implementation of depression screening and remote mental health support sessions for persons recently diagnosed with TB in Lima, Peru during the COVID-19 pandemic — R0/PR3

**DOI:** 10.1017/gmh.2024.21.pr3

**Affiliations:** London School of Hygiene & Tropical Medicine, United Kingdom of Great Britain and Northern Ireland

No accompanying comment.

# Editor report: Recommendation: Programmatic implementation of depression screening and remote mental health support sessions for persons recently diagnosed with TB in Lima, Peru during the COVID-19 pandemic — R1/PR5

**DOI:** 10.1017/gmh.2024.21.pr5

**Affiliations:** University of KwaZulu-Natal, South Africa

It is still unclear how programmatic feasibility was established. Either insert methodology and findings for assessing programmatic feasibility or remove from the abstract and main body of the paper. Please review the paper for grammatical errors.

# Editor report: Decision: Programmatic implementation of depression screening and remote mental health support sessions for persons recently diagnosed with TB in Lima, Peru during the COVID-19 pandemic — R1/PR6

**DOI:** 10.1017/gmh.2024.21.pr6

**Affiliations:** London School of Hygiene & Tropical Medicine, United Kingdom of Great Britain and Northern Ireland

No accompanying comment.

# Editor report: Recommendation: Programmatic implementation of depression screening and remote mental health support sessions for persons recently diagnosed with TB in Lima, Peru during the COVID-19 pandemic — R2/PR8

**DOI:** 10.1017/gmh.2024.21.pr8

**Affiliations:** University of KwaZulu-Natal, South Africa

No accompanying comment.

# Editor report: Decision: Programmatic implementation of depression screening and remote mental health support sessions for persons recently diagnosed with TB in Lima, Peru during the COVID-19 pandemic — R2/PR9

**DOI:** 10.1017/gmh.2024.21.pr9

**Affiliations:** London School of Hygiene & Tropical Medicine, United Kingdom of Great Britain and Northern Ireland

No accompanying comment.
